# Coculture, An Efficient Biotechnology for Mining the Biosynthesis Potential of Macrofungi via Interspecies Interactions

**DOI:** 10.3389/fmicb.2021.663924

**Published:** 2021-03-17

**Authors:** Guihong Yu, Yuman Sun, Heyang Han, Xiu Yan, Yu Wang, Xiaoxuan Ge, Bin Qiao, Lingling Tan

**Affiliations:** ^1^Shandong Province Key Laboratory of Applied Mycology, and Qingdao International Center on Microbes Utilizing Biogas, School of Life Sciences, Qingdao Agricultural University, Qingdao, China; ^2^College of Pharmacy, The Ohio State University, Columbus, OH, United States; ^3^Key Laboratory of Systems Bioengineering, Ministry of Education, School of Chemical Engineering and Technology, Tianjin University, Tianjin, China

**Keywords:** macrofungus, coculture, interspecific interaction, biosynthesis potential, metabolite

## Abstract

Macrofungi, which are also known as mushrooms, can produce various bioactive constituents and have become promising resources as lead drugs and foods rich in nutritional value. However, the production of these bioactive constituents under standard laboratory conditions is inefficiency due to the silent expression of their relevant genes. Coculture, as an important activation strategy that simulates the natural living conditions of macrofungi, can activate silent genes or clusters through interspecific interactions. Coculturing not only can trigger the biosynthesis of diverse secondary metabolites and enzymes of macrofungi, but is also useful for uncovering the mechanisms of fungal interspecific interactions and novel gene functions. In this paper, coculturing among macrofungi or between macrofungi and other microorganisms, the triggering and upregulation of secondary metabolites and enzymes, the potential medicinal applications, and the fungal–fungal interaction mechanisms are reviewed. Finally, future challenges and perspectives in further advancing coculture systems are discussed.

## Introduction

Macrofungi, most of which belong to basidiomycetes, with the minority belonging to ascomycetes, are fungi that produce spore-bearing structures visible to the naked-eye and are known for their nutritional properties, medical value, and/or degradation capacity. It is estimated that approximately 140,000 macrofungi exist in nature and now, approximately 20,000 species have been found (Zhang, 2002; Niskanen et al., 2018). Macrofungi live as haploid mycelia and grow vegetatively in soil, woods, or solid media to develop into fruiting body. The mycelium of macrofungi can also be cultivated under submerged fermentation in shaking flasks. Both the fruiting body and liquid-cultivated mycelium have been reported to produce a number of useful constituents, including bioactive lead compounds (such as alkaloids, polyketides, terpenoids, and steroids) and degradation-related oxidative enzymes (such as laccase, lignin peroxidase, and manganese peroxidase) (Qu et al., 2019; Shen et al., 2019; Zhang et al., 2019; Lira-Pérez et al., 2020), which make macrofungi an important resource for agriculture, industry, and medicine.

Currently, the genomes of more than 90 macrofungi have been sequenced, which provides great genetic knowledge for profound research (Li et al., 2018). However, the silent expression of genes or gene clusters has often been reported in macrofungi, suggesting that their potential capacities urgently need to be developed. For example, the whole-genome sequence of the widely known medicinal macrofungus *Ganoderma lucidum* indicated 16,113 annotated or predicted genes in its 43.3-Mb genome, suggesting that many gene clusters related to its metabolite or enzyme synthesis were present (Chen et al., 2012), but so far, only about 400 metabolites have been found. This meant that many genes remained cryptic or had low expression.

**Figure 1:**
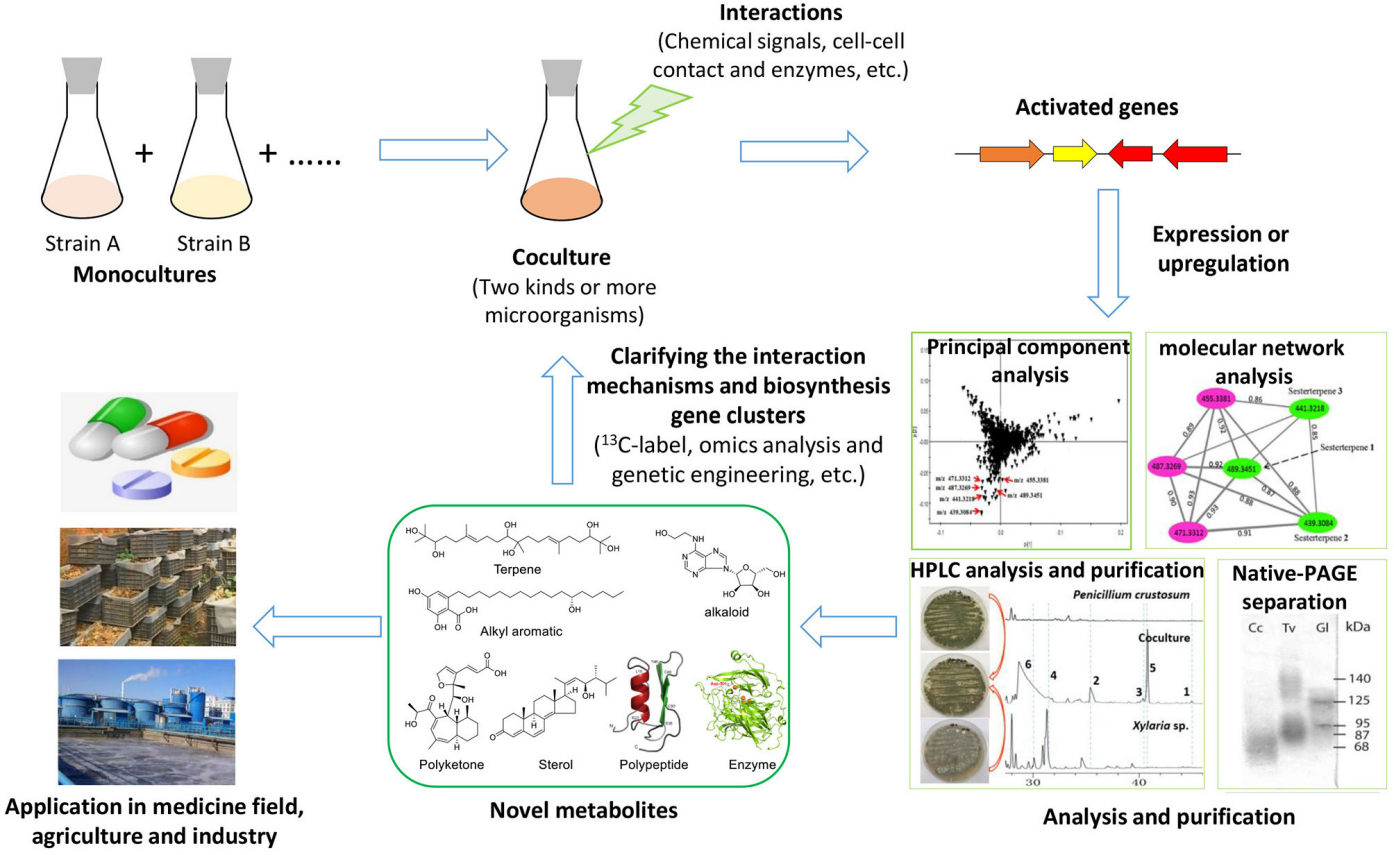
Flow chart of the coculture strategy to explore the fungal biosynthesis potential.

To explore the silent gene clusters of diverse fungi, including macrofungi, many strategies have often been used, such as the OSMAC (one strain many compounds) strategy, genetic engineering, and coculture (Scherlach and Hertweck, 2009). The OSMAC strategy, which is usually performed by altering the culture media and cultural conditions or adding the chemical inducers, is an important mean to explore the metabolic potential of fungi because of its simplicity and effectiveness, but it is difficult to produce certain types of targeted compounds in this way (Bode et al., 2002). Recently, the rapid development of biotechnology, such as the emergence of third-generation of genome sequencing and CRISPR-Cas9 (clustered regularly interspaced short palindromic repeat-Cas9) technology, has made fungal genetic manipulation for mining secondary metabolite potential (e.g., high expression of transcription factors, knockout of HDAC genes) relatively easier (Noedvig et al., 2015; Weber et al., 2015). However, the genetic system of macrofungi, especially many basidiomycetes, is much more complex than those of other fungi and is still challenging to efficiently manipulate. Only a few macrofungi, including *G. lucidum, Ganoderma lingzhi, Cordyceps militaris, Agaricus bisporus, Schizophyllum commune*, and *Coprinopsis cinerea*, have been reported to be edited by the CRISPR-Cas9 system or to achieve an overexpression system using traditional plasmids (Zhou et al., 2012; Qin et al., 2017; Sugano et al., 2017; Chen et al., 2018; Jan Vonk et al., 2019; Shen et al., 2019, 2020). By comparison, the coculture strategy can simulate the symbiotic relationship of microorganisms in nature and easily activate silenced gene clusters through the interaction between strains (Moody, 2014; Xu et al., 2018; Shen et al., 2019). Recently, coculturing has been widely used in mining unknown biosynthetic potential, such as discovering novel secondary metabolites and enzymes that might be applied in the fields of medicine, agriculture, and industry in the future (Figure 1). Further study of the interaction mechanisms in coculture systems is useful for understanding the relationship of fungal–fungal interactions in a certain ecological niche. The coculture of bacteria and filamentous fungi has been well-reviewed, but the coculture of macrofungi has not yet been reviewed in detail. In this paper, coculture among macrofungi or between macrofungi and other microorganisms, the triggering and upregulation of secondary metabolites and enzymes, the potential medicinal applications as well as the fungal–fungal interaction mechanisms are reviewed. Finally, future challenges and perspectives in further advancing coculture systems are also discussed.

## Interactions of Macrofungi in Nature

In nature, microorganisms always coexist in the same niche, and they inevitably interact with neighbors when competing for nutrients and space. These interactions can affect fungal morphologies, adaptation modes, and development patterns and trigger their ability to synthesize novel metabolites and release extracellular enzymes (Sandland et al., 2007; Bertrand et al., 2014). For macrofungi, the interactions mainly manifest as one of the relationships of antagonism, mutualism and parasitism, and sometimes, the relationships can switch from one type to another. These interactions have been used for mining the metabolic potential of fungi or for studying the relevant metabolic mechanisms.

Antagonism is the most common relationship between macrofungi living in nature, which combat via cell–cell contact or form a confrontation zone due to the production of antimicrobial substances or chemical signals, such as in relationships between *Cordyceps cicadae* and many of its endophytic bacteria (Qu et al., 2019). The appearance of confrontation zones usually indicates the induction of bioactive substances and their release to inhibit competitors. Mutualism and parasitism are also important relationships of macrofungi. For instance, the sclerotial formation of *Grifola umbellate* relies on the existence of its companion fungus *Grifola* sp., and the continuous growth and development of *G. umbellate* sclerotium requires *Armillariella mellea* as the nutrient source (Guo and Xu, 1992; Guo et al., 2001, 2002; Xing and Guo, 2003).

## Advances in the Study of Macrofungal Cocultures For Exploring the Biosynthesis Potential of Secondary Metabolites

To mimic the relationships of fungi in nature, different coculture systems, including macrofungi-macrofungi, macrofungi-microalgae, macrofungi-cyanobacteria, macrofungi-filamentous fungi, macrofungi-bacteria, and macrofungi-actinomycetes, have been established. These cocultures have shown various bioactivities, such as antimicrobial activity, antitumor activity, and antioxidant activity (Zheng et al., 2011; Shen et al., 2019).

### Cocultures Among Macrofungi or of Macrofungi With Microalgae/Cyanobacteria

Currently, the cocultures of macrofungi-macrofungi or macrofungi with microalgae/cyanobacteria are mainly performed under submerged conditions with liquid media in view of sufficient exposure and competition. Given that the growth rates of macrofungi are similar, it is proper, convenient, and common to inoculate them simultaneously. Secondary metabolites of various structural types such as phenols, terpenoids, and exopolysaccharides (EPSs) have been reported to be triggered or upregulated in these coculture systems (Zheng et al., 2011; Angelis et al., 2012).

**Figure 2:**
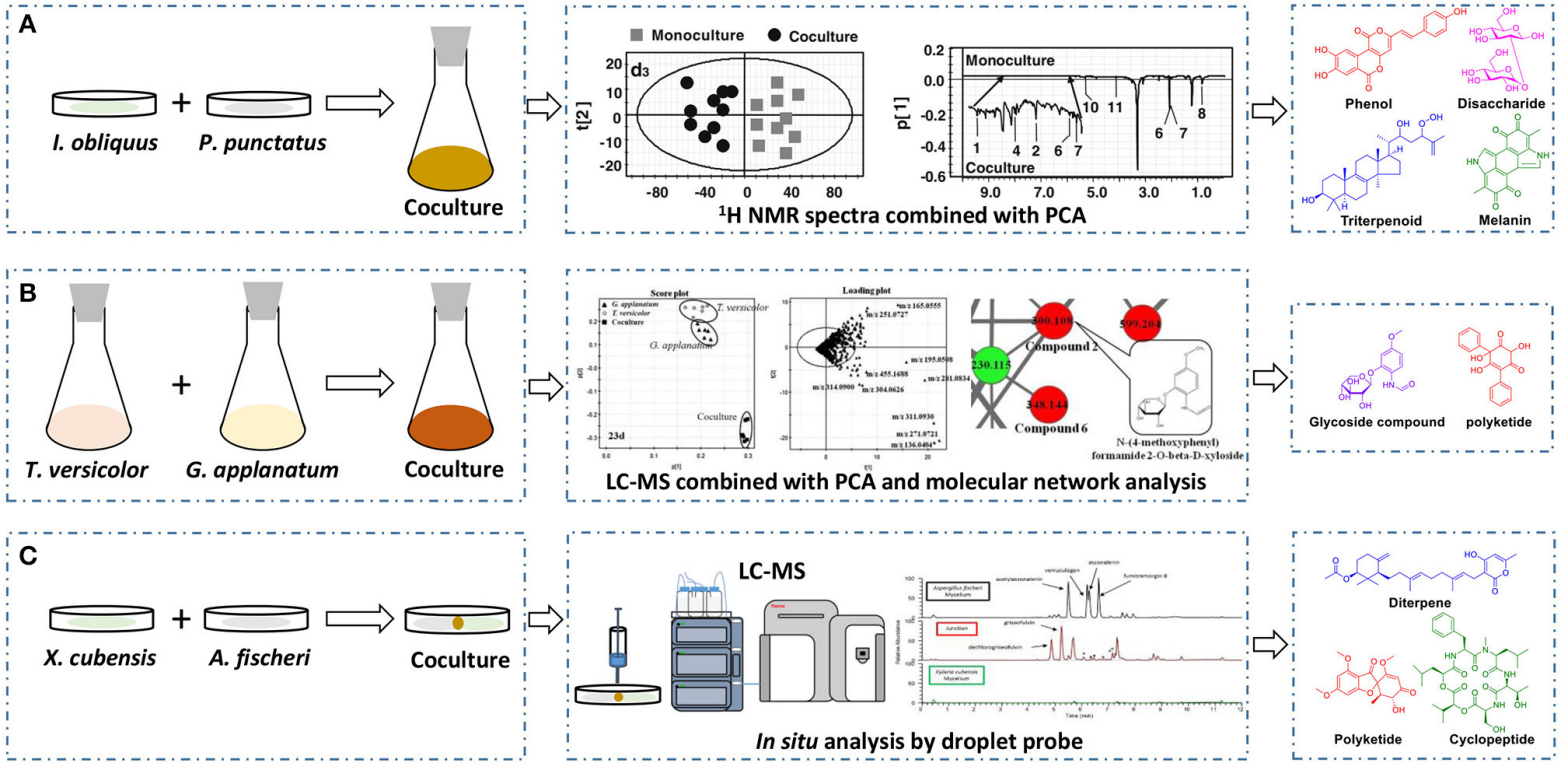
Effectively discovering newly produced or upregulated metabolites by ^1^H NMR spectra with PCA **(A)**, LC-MS combined with PCA and molecular network analysis **(B)**, or *in situ* analysis through droplet probe **(C)**.

The basidiomycete macrofungus *Inonotus obliquus* has been reported to generate diverse bioactive substances in natural habitats, but few have been reported in submerged liquid cultures. To increase its biosynthetic potential, Zheng et al. established a submerged coculture system of *I. obliquus* with another basidiomycete macrofungus *Phellinus punctatus*, resulting in the upregulation of many metabolites (Zheng et al., 2011). *I. obliquus* and *P. punctatus* were inoculated simultaneously at a ratio of 5:1 (w/w) in liquid medium, and mycelial extracts of both monocultures and coculture were analyzed by ^1^H NMR with principal component analysis (PCA) (Figure 2A). The results indicated that the upregulation of phenols [such as phelligridin C (**1**), methyl inoscavin A (**2**), and davallialactone (**3**)], triterpenoids [such as foscoparianol D (**4**) and 21,24-cyclopentalanosta-3β,21,25-triol-8-ene (**5**)], disaccharides [for instance, the new compound inotodisaccharide (**6**)], and melanins [for instance, melanin (**7**)] during the cocultivation (Table 1). Moreover, these metabolites were shown to increase the antioxidant activity and inhibitory activity against HeLa 229 cells.

**Table 1:** Typical macrofungi coculture groups and accordingly producing metabolites.

**Coculture strains**	**Coculture types**	**Upregulated or newly produced metabolites**	**Bioactivities of the metabolites**	**References**
*Inonotus obliquus* and *Phellinus punctatus*	Submerged culture in liquid medium	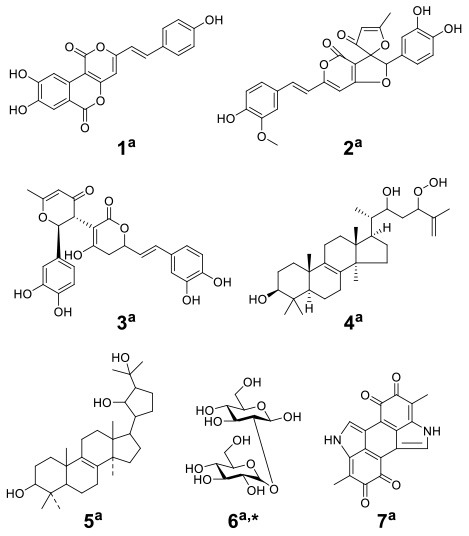	Showed the potential antioxidant activity or the inhibitory activity against HeLa 229 cells	Zheng et al., 2011
*Trametes versicolor* and *Ganoderma applanatum*	Submerged culture in liquid medium	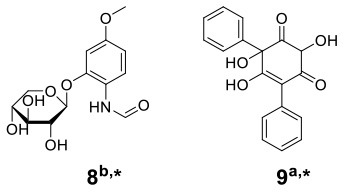	Compound **9** has potential cytotoxicity to leukemic cell line U937 and moderate antioxidant activity	Yao et al., 2016; Xu et al., 2018
*Trametes robiniophila* Murr. and *Pleurotus ostreatus*	Submerged culture in liquid medium	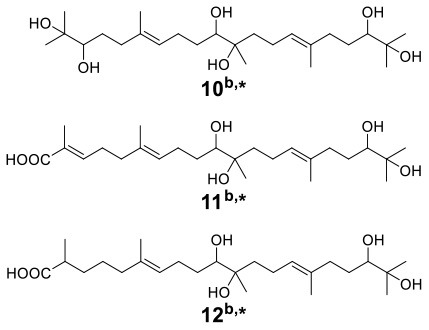	Showed potent inhibitory activities against *Candida albicans* and *Cryptococcus neoformans*	Shen et al., 2019
*Xylaria cubensis* and *Aspergillus fischeri*	Unsubmerged culture in solid medium	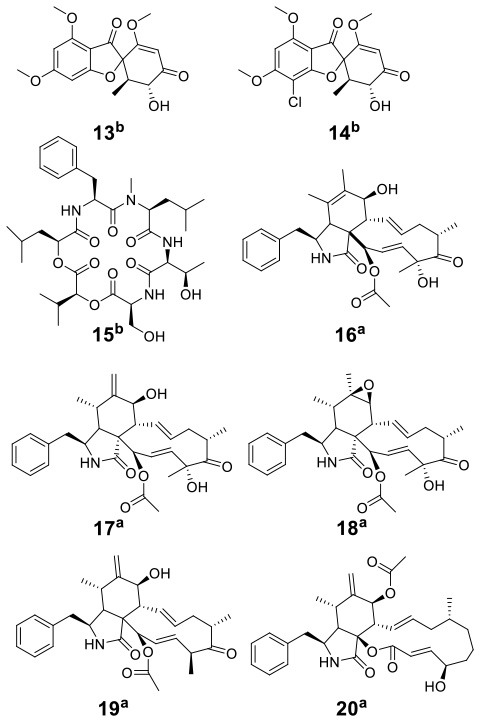	Showed fungistatic properties or cytotoxicity	Knowles et al., 2019
*Xylaria flabelliformis* and *Aspergillus fischeri*	Submerged culture in liquid medium	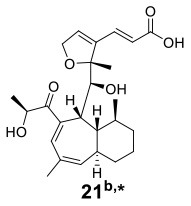	Displayed cytotoxic activity against breast, ovarian, and melanoma cancer cell lines	Knowles et al., 2020
*Xylaria* sp. with *Penicillium crustosum*	Unsubmerged culture in solid medium	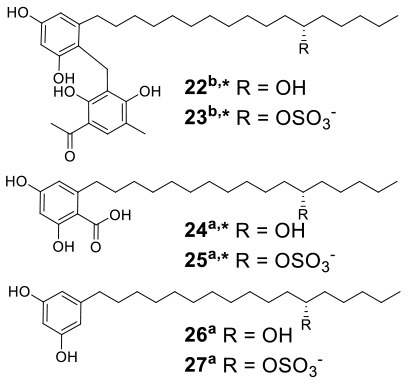	Compounds **23**, **24**, **26**, and **27** had obvious antibacterial activities	Yu et al., 2019
*Cordyceps militaris* and *Monascus rubber*	Unsubmerged culture in solid medium	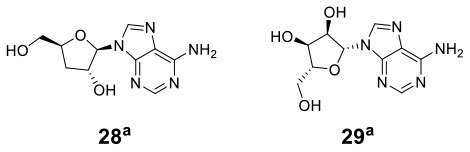	Showed the various potential activity, such as anti-tumor, anti-virus, and antimicrobial activities	Zhou and Jiang, 2008
*Serpula lacrymans* with different kinds of bacteria	Unsubmerged culture in solid medium	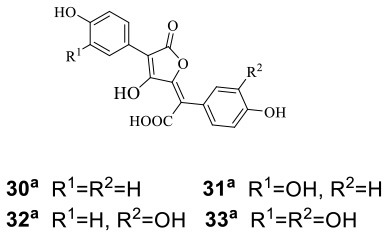	–	Tauber et al., 2016, 2018
*Pleosporales* sp. and *Bacillus wiedmannii*	Submerged culture in liquid medium	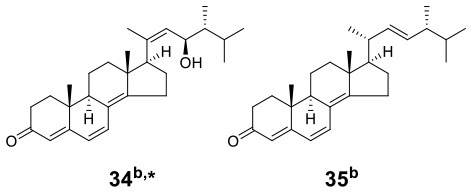	Compound **34** can inhibit *Staphylococcus aureus*	Wang et al., 2019
*Cordyceps cicadae* and its endophytic bacteria	Submerged culture in liquid medium	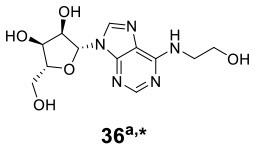	Have the potential bioactivity of enhancing immune regulation and renal function	Qu et al., 2019
*Heterobasidion abietinum* and *Streptomyces*	Submerged culture in liquid medium	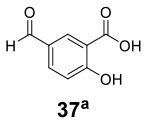	–	Keilhofer et al., 2018
Triple coculture of *Trichoderma viride, Aspergillus terreus*, and *Leptosphaerulina* sp.	Submerged culture in liquid medium	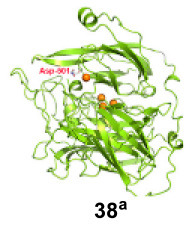	Increased the removal rate of Reactive Black 5 dye	Copete-Pertuz et al., 2019

The metabolites with “^a^” represent the upregulated compounds, the metabolites with “^b^” represent the newly produced compounds, the metabolites with “*” represent new compounds. “–” means no activities was reported.

To observe the interactions among macrofungi, Yao et al. established 136 symbiotic systems using 17 basidiomycetes (Yao et al., 2016). The coculture of *Trametes versicolor* and *Ganoderma applanatum* showed the strongest antagonistic effect, with an obvious confrontation zone on agar plate. Furthermore, they analyzed the crude extracts by LC-MS with PCA and found that 62 features were newly produced or upregulated during the coculture of *T. versicolor* and *G. applanatum* in liquid media (Figure 2B). Molecular network analysis was used to identify a new compound named *N*-(4-methoxyphenyl)formamide 2-*O*-β-D-xyloside (**8**) (Table 1), which could improve the cell viability of the human Beas-2B cell line at 1–5 μM. In addition, they found that the production of carboxylic acids such as 3-phenyllactic acid, gluconic acid, and orsellinic acid, was increased and that several glycosides were newly synthesized, including the known *N*-(4-methoxyphenyl)formamide 2-*O*-β-D-xylobioside and other undetermined glycosides. Based on these findings, they revealed a potential gene (GI: 636605689) encoding xylosyltransferases, which is responsible for glycoside production in *T. versicolor*. Moreover, the same research group found 28 additional features using optimized chromatography with PCA, leading to the production of a novel phenyl polyketide, 2,5,6-trihydroxy-4,6-diphenylcyclohex-4-ene-1,3-dione (**9**) (Table 1) with an over 15-fold increased titer in the coculture of *T. versicolor* and *G. applanatum* than monoculture of *T. versicolor* (Xu et al., 2018). Biological activity screening showed that this compound has potential cytotoxicity to the leukemic cell line U937 with a half maximal inhibitory concentration (IC_50_) of 276 ± 5 μM. Moreover, it also showed moderate antioxidant activity (82.65 ± 1.25%) at a concentration of 200 μg/ml.

**Figure 3:**
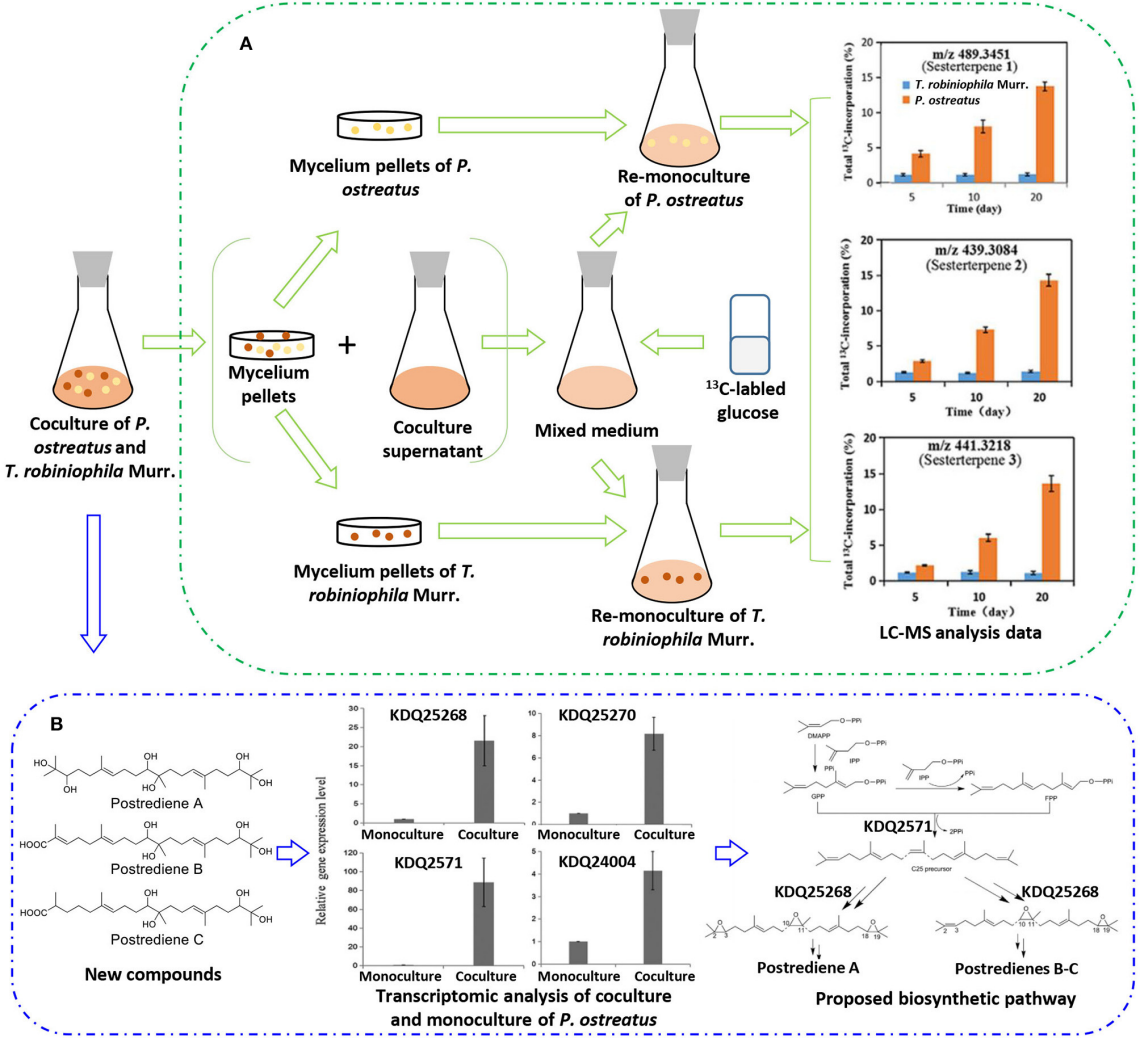
**(A)** ^13^C-labeling analysis to identify the origin of sesterterpene producers during the culture of *P. ostreatus* and *T. robiniophila* Murr. **(B)** Putative gene clusters and biosynthetic pathway of the sesterterpenes identified by transcriptomic analysis and RT-qPCR.

With the increasing drug resistance of pathogenic fungi, human-pathogenic infections have become a major threat to human safety, especially infection with *Candida albicans* and *Cryptococcus neoformans*, which show high morbidity and mortality among immunodeficient individuals. To screen out novel leading compounds with efficient antifungal activity, Shen et al. conducted 110 pairs of cocultivations of basidiomycetes. The fermentation broth from the coculture of *Trametes robiniophila* Murr. and *Pleurotus ostreatus* showed the strongest inhibitory activity against the human-pathogenic fungi *C. albicans* and *C. neoformans* (Shen et al., 2019). The combination of metabolomics analysis and activity-guided isolation led to the discovery of three unusual linear sesterterpenes, postredienes A–C (**10**–**12**) (Table 1), with 80% minimum inhibitory concentrations (MIC_80_) of 1 to 32 μg/ml against *C. albicans* and *C. neoformans*. In addition, dynamic ^13^C-labeling analysis suggested that these sesterterpenes were produced by *P. ostreatus* under the induction of *T. robiniophila* Murr. (Figure 3A). Furthermore, the results of transcriptomic analysis and RT-qPCR helped determine a putative gene cluster for biosynthesizing these novel sesterterpenes and predict potential synthetic pathways (Figure 3B). Unlike the classic biosynthesis of the C25 precursor geranylfarnesyl diphosphate (GFPP), farnesyl-diphosphate farnesyltransferase (KDQ25270) was proposed to catalyze geranyl pyrophosphate (GPP) and farnesyl pyrophosphate (FPP) to form an unusual C25 precursor. Oxidase (KDQ25268) was suggested to further modify the C25 precursor through the epoxidation reactions at positions C-2, C-3, C-10, and C-11 to generate the final sesterterpenes of postrediene A to postrediene C. This impressive study indicates a great opportunity to discover unusual second metabolites and their relevant gene clusters by the coculture strategy.

Many EPSs produced by microalgae and macrofungi can enhance immunity to resist diseases such as cancer and hepatitis. Additionally, EPS generated by lignocellulolitic fungi is also of great importance in wood decay. Angelis et al. investigated the production of EPS in coculture of the basidiomycete *T. versicolor* and *Agaricus blazei*, as well as the cocultures of *T. versicolor* or *A. blazei* with microalgae and cyanobacteria in liquid fermentation (Angelis et al., 2012). The hydrosoluble EPS synthesized by monocultures and cocultures were compared using ^13^C-NMR and GC-MS. The results indicated that the cocultures could not only obviously enhance the production of EPSs and reduce the fermentation time, but could also trigger the production of new polysaccharides. Although structural characterization of the polysaccharides requires further interpretation, this study provides a new strategy to improve the total production of EPSs and to activate new EPSs synthesis for medical and/or industrial applications.

### Cocultures of Macrofungi and Filamentous Fungi

To explore the biosynthetic potential of secondary metabolites by coculturing of macrofungi and filamentous fungi, most studies have been carried out by cultivating the mycelium on solid media. Currently, a number of secondary metabolites, such as mycotoxins, alkyl aromatics, and nucleosides, have been reported to be synthesized through activation in these coculture systems (Zhou and Jiang, 2008; Yu et al., 2019; Knowles et al., 2020).

Knowles et al. cocultured *Xylaria cubensis* with *Aspergillus fischeri*, which can produce griseofulvin and a suite of mycotoxins, respectively (Knowles et al., 2019). The authors investigated the spatial distribution of the fungal secondary metabolites on solid medium in monocultures and coculture using a droplet-liquid microjunction-surface sampling probe (droplet probe; Figure 2C). For example, they found that the two compounds griseofulvin and dechlorogriseofulvin were only detected at the contact zone of the coculture but existed throughout the mycelia in monoculture of *X. cubensis* and showed more accumulation at the colony edge. Further large-scale fermentation revealed that the coculture of *X. cubensis* and *A. fischeri* not only triggered the production of 5′-hydroxygriseofulvin (**13**), dechloro-5′-hydroxygriseofulvin, (**14**), and hirsutatin A (**15**) but also increased the titers of cytochalasin C (**16**), cytochalasin D (**17**), cytochalasin Q (**18**), zygosporin E (**19**), and 7-*O*-acetylcytochalasin B (**20**) (Knowles et al., 2019; Table 1). Moreover, they found a new compound, wheldone (**21**) (Table 1), which displayed cytotoxic activity against breast, ovarian, and melanoma cancer cell lines (Knowles et al., 2020). To explore the roles of secondary metabolites in interspecific interactions, they further established another cocultured system—*X. cubensis* with a mutant strain of *A. fischeri* that lacked the master regulator *lae*A in secondary metabolism. The mutant *A. fischeri* was found to be displaced by *X. cubensis* because it was unable to regulate secondary metabolite biosynthesis in the competition of the coculture (Knowles et al., 2019). Similarly, Yu et al. researched another *Xylaria* species by coculturing (Yu et al., 2019). In this experiment, four novel alkyl aromatics, penixylarins A-D (**22**–**25**) and two known compounds, 1,3-dihydroxy-5-(12-hydroxyheptadecyl)benzene (**26**) and 1,3-dihydroxy-5-(12-sulfoxyheptadecyl)benzene (**27**), were isolated when *Xylaria* sp. HDN13-249 was cocultured with *Penicillium crustosum* PRB-2 on solid medium (Table 1). Further analysis of the LC-MS results and the structural characteristics indicated that novel compounds **22** and **23** were formed in the coculture by a non-enzymatic Michael addition reaction between two fragments from *P. crustosum* PRB-2 and *Xylaria* sp. HDN13-249, respectively, while compounds **24**–**27** could be synthesized in monoculture of *Xylaria* sp. HDN13-249, but their titers were obviously enhanced in the coculture. These findings suggested that different mechanisms appeared to produce new compounds in the coculture system. Evaluation of the activities showed that compounds **23**, **24**, **26**, and **27** could inhibit bacterial growth, among which compound **24** displayed the best activity against *Mycobacterium phlei*, with an MIC of 6.25 μM. This result suggested that compound **24** could be designed as a potential antituberculosis leading drug.

The macroascomycete *C. militaris* is a traditional Chinese medicine that produces a variety of bioactive components, such as adenosine and cordycepin. However, these active components usually have an issue of low production titers and high production costs. Researchers are attempting to solve this challenge through a coculture strategy. Zhou and Jiang cocultivated *C. militaris* CM9-26 and *Monascus rubber* MT305 on an optimized solid medium in an orthogonal test, resulting in the significant enhancement of many bioactive components (Zhou and Jiang, 2008). The titers of cordycepin (**28**) and adenosine (**29**) were 2.5- and 5.1-fold higher than those in the monoculture of *C. militaris* CM9-26, respectively (Table 1). The pigments produced by *M. rubber* MT305 were 3-fold higher than those produced by its monoculture. Similarly, *C. militaris* was cocultured with *M. rubber* in an optimized solid medium, temperature and humidity. The titer of cordycepin (**28**) was increased by up to 1.4-fold of that in the monoculture of *C. militaris*, and its fruit-body production was increased as well (Liu, 2015; Table 1).

### Cocultures of Macrofungi and Bacteria or Macrofungi and Actinomycetes

The cocultures of macrofungi and bacteria have been studied more extensively compared with other cultivated pairs. Both submerged liquid cultivations and solid cultivations have been investigated by adjusting the inoculation quantity and inoculation times, which induce various constituents such as antimicrobial peptides, pigments, and ergosterol (Essig et al., 2014; Tauber et al., 2016; Wang et al., 2019).

The basidiomycete macrofungus *C. cinerea* grows on the dung of herbivores, which provides a high humidity habitat with diverse microbes. To mimic this habitat and identify new antibiotics and fungicides, Essig et al. studied the interaction of *C. cinerea* with different bacteria in an artificial coculture system. *C. cinerea* was first grown on glass beads submerged in liquid medium, and after 60 h, this medium was replaced by a culture solution of bacteria including *Bacillus subtilis* 168, *Pseudomonas aeruginosa* 01, or *Escherichia coli* BL21 (Essig et al., 2014). *C. cinerea* was found to secrete some constituents to inhibit the growth of *B. subtilis* 168. Further study led to the identification of a novel antimicrobial peptide, copsin. Copsin showed broad inhibitory activity against diverse gram-positive bacteria, including human-pathogenic *Enterococcus faecium* and *Listeria monocytogenes*, through the mechanism of binding specifically with peptidoglycan precursor lipid II to interfere with the biosynthesis of the bacterial cell wall.

Atromentin has been proven to play a role in redox cycling, but there are still no clues about the function of this pigment in the interaction of natural systems. To solve this mystery, Tauber et al. established cocultured systems of the model basidiomycete *Serpula lacrymans* with many terrestrial bacteria, including *Bacillus subtilis* 3610, *Pseudomonas putida*, and *Streptomyces iranensis*, on solid medium. During cocultivation, *B. subtilis* 3610 and *P. putida* were precultured overnight, while *S. iranensis* was precultured for 4 days before spotting onto the fungal mycelial bed precultured for 13–14 days (Tauber et al., 2016). As a result, the gene cluster related to atromentin biosynthesis, including atromentin synthetase and aminotransferase was upregulated, and the corresponding atromentin-derived pigments, i.e., atromentic, xerocomic, isoxerocomic, and variegatic acids (**30**–**33**) (Table 1), were increased. Further bioinformatics suggested that highly conserved motifs of atromentin synthesis genes might be regulated by similar transcription factors through the same regulatory mechanisms. In addition, although these pigments had no antimicrobial activity, they might play a role in the nutritional perspective and be related to the redox cycling of lignocellulose degradation. Thus, they could be induced by a nutritional response or coregulated along with other cellular processes, such as stress and secondary metabolism. In 2018, the same research group further investigated this phenomenon in depth, and they demonstrated that coculturing of *S. lacrymans* with 10 other bacteria (such as *Lysinibacillus fusiformis* M5, *Arthrobacter* spp., and *Micrococcus luteus*) could also improve the production of atromentin-derived pigments (Tauber et al., 2018). The exogenous addition of protease inhibitors and the use of heat-killed bacteria in coculture suggested that enzymatic hyphal damage and/or accordingly released peptides could trigger the synthesis of atromentin-derived pigments.

To diversify the synthesis of secondary metabolites of the endophytic macroascomycete *Pleosporales* sp. F46 isolated from *Mahonia fortunei*, Wang et al. treated *Pleosporales* sp. F46 with *Bacillus wiedmannii* Com1, which was an endophytic bacterium isolated from the same plant (Wang et al., 2019). In this coculture system, the bacterium *B. wiedmannii* Com1 was precultured for 3 days and then added to the coculture medium (solid rice medium). After 6 days, *Pleosporales* sp. F46 cells precultured for 7 days were added to the same coculture medium. By cocultivation, two new HPLC peaks were observed in the crude extracts, which were not detected in the individual monocultures. Further chemical structural analysis led to the identification of a new ergosterol derivative, 23*R*-hydroxy-(20*Z*,24*R*)-ergosta-4,6,8(14),20(22)-tetraen-3-one (**34**), and a known related compound, (22*E*,24*R*)-ergosta-4,6,8(14),22-tetraen-3-one (**35**) (Table 1). Compound **34**, with the MIC of 100 μg/ml against *Staphylococcus aureus*, was the first reported ergosterol derivative containing a side chain with a Δ^20(22)^-double bond.

*Cordyceps cicadae* as an ascomycete fungus is parasitic in the larvae of *Cicada flammata* and can produce diverse compounds such as *N*6-(2-hydroxyethyl)-adenosine (HEA) (**36**) (Table 1), adenosine, ergosterol, and cordycepin to improve immune regulation and renal function. Fifty-four endophytic bacteria were isolated along with *C. cicadae*, and four of them were chosen to be cocultured with *C. cicadae* according to metabolite analysis and their inhibitory phenomena (Qu et al., 2019). Coculture was performed with *C. cicadae* precultured for 5 days before inoculating bacteria (including *Enterobacter aerogenes* T4-4, *Serratia marcescens* T4-16, *Cedecea neteri* T4-8, and T4-11). Further metabolite profiling indicated that the titer of HEA was increased to at least 13-fold compared to that in the monoculture. Meanwhile, three nucleosides (i.e., adenosine, uridine, and guanosine), as the structural analogs of HEA, were found to be decreased significantly during the coculture.

There is little research on the cocultures of macrofungi and actinomycetes. One example is the coculture of *Heterobasidion abietinum* and *Streptomyces. Heterobasidion* spp. can cause root rot in *Norway spruce*, and its infection is further intensified by rhizospheric streptomycetes. To clarify their interaction mechanism, Keilhofer et al. studied the coculture system of *H. abietinum* 331 and *Streptomyces* AcH 505, including the mode of action on *Norway spruce* (Keilhofer et al., 2018). *Streptomyces* AcH 505 and *H. abietinum* 331 began to be cocultured in liquid medium after the monocultivation for 2 and 7 days. HPLC analysis showed that 5-formylsalicylic acid (5-FSA) (**37**) produced by *H. abietinum* 331 was upregulated under the stimulation of *Streptomyces* AcH 505 (Table 1). *H. abietum* 331 cocultured with many other *Streptomycetes* isolated from soil had similar results. *Norway spruce* seedlings treated with 5-FSA indicated that ergosterol in infected roots was obviously increased, and the infection degree was comparable to that of the seedlings treated by *H. abietinum* 331 and *Streptomyces* AcH 505. Further research demonstrated that 5-FSA can interfere with the emission of defensive signals in *Arabidopsis thaliana* in a manner similar to salicylic acid.

## Advances in the Study of Macrofungal Cocultures for Exploring the Biosynthesis Potential of Functional Enzyme

With continuous agricultural and industrial development, the environmental pollution caused by crop straw burning, petroleum hydrocarbon (PHC) overexploitation, and dye and pesticide abuse is emerging as a serious problem. Many macrofungi can produce various oxidative and degradative enzymes, such as laccase (**38**) (Table 1), lignin peroxidase, and manganese peroxidase. These enzymes not only have great potential in the treatment of agricultural and industrial wastes enriched with lignocellulose, but can also be used in industrial production, such as pulp delignification and bleaching, dye decolorization, sewage disposal, and food processing. Researchers have found that the coculture strategy can dramatically enhance the production of oxidative and degradative enzymes compared to the monoculture, including coculturing among macrofungi (such as *P. ostreatus* with *T. versicolor* and *Marasmius pallescens* with *Marasmiellus troyanus*), coculturing of macrofungi and filamentous fungi (such as *T. versicolor* with *Trichoderma harzianum*/*Candida* sp./*Aspergillus niger, Lentinula edodes* with *Trichoderma* sp., *P. ostreatus* with *Trichoderma longibrachiatum*, and *C. cinerea* with *Gongronella* sp.), and coculturing macrofungi and bacteria (such as *Fomitopsis pinicola* with *B. subtilis*) (Freitag and Morrel, 1992; Savoie et al., 1998; Baldrian, 2004; Velázquez-Cedeño et al., 2004; Ferreira Gregorio et al., 2006; Zhang et al., 2006; Chi et al., 2007; Stoilova and Krastanov, 2008; Flores et al., 2009; Wang et al., 2009; Hiscox et al., 2010; Wei et al., 2010; Chen et al., 2011; Kuhar et al., 2015; Ma and Ruan, 2015; Mewada et al., 2017; Huo et al., 2019; Kumar et al., 2019; Wiberth et al., 2019; Lira-Pérez et al., 2020). For example, the production of laccase in the coculture of *T. versicolor* and *T. harzianum* was more than 40-fold higher than that in the monoculture (Baldrian, 2004).

### Cocultures Among Macrofungi

To enhance the production of functional enzymes, researchers have tried to set up cocultures among macrofungi in either liquid media or solid media, both of which have obtained good results with the increment of oxidative and degradative enzymes, such as P450 monooxygenases, dioxygenases, and laccases (Yanto and Tachibana, 2014).

Petroleum hydrocarbons (PHCs) and related products released into the environment during the exploitation, processing, and utilization of petroleum are carcinogenic and mutagenic pollutants that are seriously harmful to the health of humans and animals. An increasing number of studies have reported that microbial coculture can biodegrade PHCs more efficiently. Yanto and Tachibana studied how to efficiently degrade PHCs through the cocultures of different basidiomycetes, including *Trametes versicolor* U97, *Pleurotus ostreatus* PL1, *Cerena* sp. F0607, and *Polyporus* sp. S133 with ascomycetes *Pestalotiopsis* sp. NG007 (Yanto and Tachibana, 2014). The influence of different inoculation proportions was investigated for four pairs of these strains. The results showed that a 50/50 inoculation proportion of *Polyporus* sp. S133 and *Pestalotiopsis* sp. NG007 had higher biodegradation rates of PHCs. For example, the degradation rate of the main aromatic ingredients in PHCs increased from 53.06% (the monoculture of *Pestalotiopsis* sp. NG007 for 15 days) and 37.40% (the monoculture of *Polyporus* sp. S133 for 15 days) to 89.35% in this coculture for 15 days. *In vitro* crude enzymatic assays and inhibition experiments suggested that the increased production of degradative enzymes, such as P450 monooxygenases, dioxygenases, and laccases, led to the high biodegradation of PHCs.

Indigo carmine is a traditional dye that widely used in dyeing plastic and textiles. The methods of removing indigo carmine from polluted water include absorption, photocatalysis, and biodegradation. Among those methods, the absorption method is low cost and easy to operate. For instance, sesame straw, which is an abundant agricultural waste, can be used as an absorbent. Because the absorbance efficiency of sesame straw is limited (21.90 mg/g), Li et al. improved it by coculturing *Tremella fuciformis* and *Morchella* sp. with sesame straw (Li et al., 2019). The absorption efficiency of sesame straw fermented by the cocultured strains was increased nearly 2-fold compared with untreated or *Morchella* sp.-treated sesame straw and was up to 1.4-fold compared with *T. fuciformis*-treated sesame straw. Moreover, with the addition of inductors including acetic acid, Cu^2+^, and alkaline lignin during fermentation by the coculture, the absorption of fermented sesame straw was further increased to 74.25 mg/g.

Malachite green (MG) is a triphenylmethane dye and antifungal agent that also shows high toxicity toward the health of mammals. In 2015, Kuhar et al. found that culturing *G. lucidum* and *T. versicolor* simultaneously in a sawdust-based medium resulted in increased laccase production by 3.5- and 9.2-fold than *G. lucidum* and *T. versicolor* in the monoculture. This finding inspired them to investigate the MG degradation ability of the coculture system. The results showed that the time required to completely decolorize MG in the coculture system was obviously decreased, and the half-life of decolorizing MG was approximately 1/3 that of the monocultures of *G. lucidum* and *T. versicolor* (Kuhar et al., 2015).

Similarly, cibracron brilliant red 3B-A dye is also widely used in textile industries and is harmful to the environment and challenging to address. In 2018, Bankole et al. investigated the dye-degrading abilities of two ascomycete white-rot macrofungi, *Daldinia concentrica* and *Xylaria polymorpha* under the monoculture and coculture (Bankole et al., 2018). They found that coculturing *D. concentrica* and *X. polymorpha* by solid state fermentation could remove dye more efficiently than the monocultures of either strains. When cibracron brilliant red 3B-A dye was treated with the monoculture of *X. polymorpha*, the absorbance peak was deceased more efficiently than when treated with *D. concentrica*, but only less than half of the dye was degraded. In contrast, when treated with *D. concentrica* and *X. polymorpha* coculture, almost 100% of dye was degraded. In addition, phytotoxicity evaluation indicated that the degradation products of the cibracron brilliant red 3B-A dye produced by the coculture of *D. concentrica* and *X. polymorpha* became less toxic.

### Cocultures of Macrofungi and Filamentous Fungi or Macrofungi and Bacteria

Lignocellulosic resources, such as agricultural residues, waste woods, and paper, are promising low-cost feedstocks for producing renewable energy. For example, to improve corn stover bioconversion, Ma and Ruan established an effective lignocellulose-biodegrading system by coculturing of *Coprinus comatus* and *Trichoderma reesei* in liquid medium (Ma and Ruan, 2015). Their research showed that the production of lignocellulolytic enzymes such as carboxymethyl cellulase, xylanase, and laccase in the coculture was much higher than that in the monocultures, and the lignocellulose delignification times were becoming shorter. To further improve the production of lignocellulolytic enzymes in the coculture system, they also investigated the influences of chemical inducers, metal ions, inoculation ratios, and inoculation intervals. The results suggested that those factors all have obviously influences, among which, with an inoculation ratio of *C. comatus* and *T. reesei* of 5:2 and an inoculation interval of 12 h, the production of laccase was increased 2.6-fold and the maximal production was obtained 3 days earlier, compared to the monoculture of *C. comatus*. In addition, the influences of the temperature, pH, and solid/liquid ratio on the delignification and saccharification of corn stover were also tested. The results suggested that the temperature of 50°C, the pH of 5.0, and the solid/liquid ratio of 1:20 were the optimal conditions, and a maximum delignification rate of 66.5% and the conversion rate of original polysaccharides to fermentable sugars of 82.2% were obtained at 50°C. This study provided a useful strategy for transforming corn stover into sugar by simultaneous biodelignification and saccharification.

Benzo[a]pyrene (BaP), which is a carcinogenic and mutagenic polycyclic aromatic hydrocarbon that exists in coal tar from the smoke of car exhaust, tobacco, and wood burning, etc., has been classified as a pollutant prior to treatment (Bhattacharya et al., 2017). Bhattacharya et al. focused on the treatment of BaP by cocultures of *P. ostreatus* PO-3 with bacteria or non-basidiomycete fungi. *P. ostreatus* PO-3 was precultured in liquid medium for 15 days and then cocultured with either the filamentous fungi *A. niger, Penicillium chrysogenum*, and *T. reesei* or the bacteria *P. aeruginosa* and *Bacillus cereus*. Among these cocultures, cocultures of *P. ostreatus* PO-3 with *Penicillium chrysogenum* MTCC 787 or with *Pseudomonas aeruginosa* MTCC 1688 showed degradation rates at 86.1 and 75.1%, respectively, which were higher than that of the monoculture of *P. ostreatus* PO-3 (64.3%). Researchers proposed that the improvement of BaP degradation in fungal cocultures was related to the increased production of both lignolytic and non-lignolytic enzymes such as laccase, cytochrome P-450 monooxygenase, and epoxide hydrolases. The enhanced BaP degradation in the basidiomycete and bacterial cocultures was probably associated with bacterial surfactants and/or other bacterial enzymes such as salicylate hydroxylase, 2-caroxybenzaldehyde dehydrogenase, and catechol 1, 2-dioxygenase. In this study, BaP was degraded into polar and hydrosoluble products which could be further mineralized by other indigenous microorganisms.

In 2019, Copete-Pertuz et al. observed the production of ligninolytic enzymes with decolor ability in the ascomycete white-rot fungus of *Leptosphaerulina* sp. by coculturing with other microorganisms, including the species *Aspergillus, Trichoderma, Fusarium*, and *Penicillium* (Copete-Pertuz et al., 2019). Among these, *Trichoderma viride* and *Aspergillus terreus* showed the best inducing effect in solid media assays. Then, a triple-coculture system was established by response surface methodology, which was performed by inoculating *T. viride* (1,000 μl) and *A. terreus* (1,000 μl) into 7-day precultured *Leptosphaerulina* sp. in liquid medium. The amounts of laccase, versatile peroxidase, and manganese peroxidase were found to be increased by 8-, 36-, and 88-fold, respectively. The removal rate of Reactive Black 5 dye was increased by 1.2-fold compared with the monoculture of *Leptosphaerulina* sp.

These studies indicated the efficiency of macrofungal coculture in the eco-friendly restoration of polluted environments, providing an important direction for further research on treating organic pollution.

## Application of Macrofungi Cocultures for Generating the Fruiting Body

With the increasing demand for edible and medicinal macrofungi, artificial cultivation is becoming increasingly common. However, researchers found that the fruiting bodies of some macrofungi were difficult to cultivate under the standard laboratory conditions, partly because of the lack of symbiotic microorganisms. Thus, the coculture strategy mimicking the natural environment was developed for fruiting body formation of these edible and medicinal macrofungi on solid media (Guo et al., 2001; Liu, 2015).

*Grifola umbellate*, which is a medicinal macrofungus whose sclerotium has the effect of diuresis and detumescence, is hard to artificially cultivate before researchers identified the symbiotic relationship with *Grifola* sp. (companion fungus of *G. umbellate*) and *A*. *mellea*. As mentioned earlier in this paper, Guo et al. conducted a systematic study on the cocultures of *G. umbellate, Grifola* sp., and *A*. *mellea* (Guo and Xu, 1992; Guo et al., 2001, 2002; Xing and Guo, 2003). The results showed that *G. umbellate* could produce a mass of mycelium bundles on the surface of its colony when cocultured with its companion fungus *Grifola* sp. This was a phenomenon in the early stage of sclerotial formation, while almost no mycelium bundles was formed in the monoculture. *A. mellea* was found to infect *G. umbellate* after the sclerotium formation, and the following nutrient exchange could promote the sclerotium growth.

In addition to the example mentioned above, the coculture strategy can also improve the quality of the fruit body. For instance, Liu et al. found that the active constituent of cordycepin in the fruit body of *C. militaris* was enhanced by coculturing with *M. rubber* (Liu, 2015).

## Interaction Mechanisms in Macrofungi Cocultures

Elucidation of the potential interaction mechanisms is important for improving the application of coculture strategy. Generally, microorganisms are considered to communicate with each other through either chemical signals or cell–cell contact to change the production of related metabolites. In recent years, an increasing number of experiments have been carried out to demonstrate the interaction mechanisms, such as exogenous addition experiments, ^13^C-labeling analysis, proteomic analysis, and genetic engineering.

### Chemical Signals Mediating the Interactions in Cocultures

To compete for limited nutrition and living space, the microorganisms in the coculture system often produce diverse chemical signals to communicate with each other. These chemical signals can affect both cell growth and secondary metabolite production.

Ferreira-Gregorio et al. reported the rapid induction and release of laccase and manganese peroxidase into the culture supernatant when coculturing *M. pallescens* and *M. troyanus* in liquid medium. Neither enzyme was produced in the monocultures (Ferreira Gregorio et al., 2006). Further research showed that laccase could be induced to different degrees when adding the filter-sterilized or autoclaved supernatant of *M. pallescens* into *M. troyanus* culture, but no effect occurred in the opposite experiment. Both *M. troyanus* and *M. pallescens* (in a small quantity) were able to produce laccase when using the inducer 2,5-dimethylalanine. These results suggested that *M. pallescens* could secrete thermostable and thermolabile chemical signals to induce the laccase produced by *M. troyanus*. Huo et al. discovered that *C. cinerea* grew slowly and produced little laccase in a monoculture with sucrose as the carbon source, but the production of laccase was significantly increased in the coculture with *Gongronella* sp. w5 (Huo et al., 2019). Further study showed that strain w5 could hydrolyze sucrose to glucose and fructose during coculture, and fructose was an efficient carbon source for *C. cinerea* growth and laccase production. In addition, isolating strain w5 with *C. cinerea* by dialysis tubes still led to laccase activity similar to that of the coculture treatment, suggesting that certain extracellular metabolites, as chemical signals, were also responsible for the increased production of laccase. Indeed, they found that the ethyl acetate extract of strain w5 could also increase the production of laccase in the monoculture of *C. cinerea*. *p*-Hydroxybenzoic acid was isolated from this extract, which could improve laccase production to a level similar to that of the coculture by using fructose as the carbon source.

As mentioned previously, Shen et al. discovered three novel sesterterpenes by coculturing of *P. ostreatus* and *T. robiniophila* Murr. They further applied dynamic ^13^C-labeling analysis to find that ^13^C-labeled sesterterpenes could only be produced when *P. ostreatus* was stimulated by *T. robiniophila* Murr. (Figure 3A). By further analyzing the ^13^C-labeling patterns in the monoculture of *P. ostreatus* with the addition of the supernatant of the coculture, they speculated that the biosynthesis of sesterterpenes was triggered by some chemical signals released into the culture medium.

In addition, Knowles discovered that *X. cubensis* could produce more fungistatic compounds when cocultured with *A. fischeri*. Meanwhile, *A. fischeri* increased the titer of the mycotoxins. Furthermore, they explored the functions of secondary metabolites during the interaction by constructing a mutant *A. fischeri* lacking the master regulator *laeA* of secondary metabolism and coculturing it with *X. cubensis* (Knowles et al., 2019). The mutant *A. fischeri* was found to be displaced by *X. cubensis* because it lost the ability to regulate secondary metabolite biosynthesis in this competition system.

### Cell–Cell Contact, Enzymes, or Related Proteins Mediating the Interactions in Cocultures

In addition to chemical signals, the enzymes or related proteins produced by cocultured microorganisms can also affect the metabolic pathways. Kumar et al. studied the interaction mechanism between *Trametes ljubarskyi* and *Rhodotorula mucilaginosa*, which was a coculture system that can efficiently produce laccase (Kumar et al., 2019). Investigation by electron microscopy indicated that the yeast *R. mucilaginosa* adhered to the mycelia of *T. ljubarskyi*, and the strong interactions resulted in morphologic changes in the yeast and mycelial surface damage of *T. ljubarskyi*. Further proteomic analysis indicated that the differential synthesis of a series of oxidoreductases, antioxidants, membrane-related proteins, and transporter proteins would be beneficial for the cosurvival of these two fungi.

As mentioned earlier in this paper, Tauber et al. found that the coculture of *S. lacrymans* with different bacteria increased the production of atromentin-derived pigments (Tauber et al., 2018). Further exogenous addition experiments showed that the addition of cell-wall-damaging enzymes (i.e., lytic enzymes and proteases) into *S. lacrymans* can also increase the production of those pigments, but other lysozymes or mechanical damage did not obtain the same effect. In addition, pigment production was obviously reduced when *S. lacrymans* was treated with heat-killed bacteria or by protease inhibitors and *B. subtilis* simultaneously. These results suggested that enzymatic hyphal damage and/or released peptides could promote the titers of atromentin-derived pigments.

### Carbon Source Succession Mediating the Interactions of Cocultures

In the coculture systems, microorganisms compete with limited nutritions, such as carbon and nitrogen sources for survival, among which researchers demonstrate that carbon source succession is an important factor in the regulation of metabolic pathways. In 2009, Wang et al. found that laccase activity was increased to 10,500 ± 160 U/L by coculturing *T. versicolor* and *Candida* sp. HSD07A, 11.8-fold higher than that of the monoculture of *T. versicolor* (Wang et al., 2009). Enzymatic analysis of *Candida* sp. HSD07A suggested that it could not produce laccase but could excrete amylase and cellulase which can hydrolyze the cell walls of *T. versicolor*. Similar results have also indicated that amylase and cellulase could induce the production of pigments in *Monascus* by hydrolyzing its cell walls (Shin et al., 1998). Thus, Wang et al. speculated that the upregulation of laccase would be related to the effect of amylase and cellulase on the cell walls of *T. versicolor*. However, they noticed that the hydrolysis of cell walls or the addition of enzyme solutions could not result in the upregulation of laccase, suggesting that the hydrolysis of cell walls was not the key reason. Furthermore, researchers explored whether glucose starvation could lead to the upregulation of laccase in *T. versicolor* due to the high ability of *Candida* sp. HSD07A in assimilating glucose. This hypothesis was preliminarily supported by analyzing the relationship between the sugar amount and laccase activity.

Similarly, the mechanism of upregulating laccase by coculturing *G. lucidum* and *Candida* sp. HSD07A was studied by Li et al. (2010). This study revealed that the nitrogen source, sulfur source, hydrolytic enzymes, and inducers did not have obvious effects on laccase production, and glucose deprivation could only result in the upregulation of laccase to some extent. NMR and GC data indicated that *Candida* sp. HSD07A could transform glucose into glycerol and ethanol, among which glycerol was an effective carbon source for *G. lucidum*. Further glycerol addition experiments suggested that the production of glycerol was indeed another key reason for the upregulation of laccase in addition to glucose starvation. This was likely because glycerol could prolong the production time of laccase under the glucose starvation state. These studies demonstrate that the glucose starvation and glycerol succession that result from yeast *Candida* sp. HSD07A are new approach to improve laccase production in basidiomycete fungi.

## Conclusion and Perspectives

Compared with the complicated living conditions in nature, many genes or clusters of microorganisms are silent in standard culture under the laboratory conditions. To activate these silent genes or clusters, the coculture strategy is considered as an efficient approach, because it can mimic the symbiotic relationships of microorganisms in their intrinsic habitats (Moody, 2014; Xu et al., 2018; Shen et al., 2019). Secondary metabolites induced by cocultures, such as small molecular metabolites, polysaccharides, and polypeptides, usually display diverse bioactivities, including antimicrobial activity, antitumor activity, and antioxidant activity, and represent a potential and powerful resource to discover the leading compounds for medicinal development. Moreover, the cocultures of macrofungi can improve the production of oxidative and degradative enzymes, such as laccase, lignin peroxidase, and manganese peroxidase, which is very useful in agricultural and industrial wastes biodegradation, pulp delignification and bleaching, dye decolorization, sewage disposal, and food processing.

Notably, with in-depth coculture research, the detailed interaction mechanism is becoming a hot field for exploration but is still a mystery for researchers. Macrofungi cultivated in a given coculture system can communicate with each other by producing signal metabolites to reach an equilibrium relationship. This interaction process during communication is complicated and is often associated with more than one factor. A number of strategies, including exogenous addition experiments, dynamic ^13^C-labeling analysis, proteomics, and genetic engineering, have been used, but the interaction mechanisms of most coculture systems have not been clarified. To further elucidate the mechanism, more approaches should be developed, for example, multi-omics technology and CRISPR-Cas9-based genetic editing, which will be helpful to confirm the functions of chemical signals, to identify genes and clusters for secondary metabolite biosynthesis and to determine the regulatory mechanisms. This understanding will in turn help simplify the culture conditions, such as by the addition of certain inducers, to achieve a similar or even better effect of that in the coculture. Overall, with the progress of macrofungal genetic manipulating tools, multi-omics technology and a deeper understanding of the interaction mechanisms, the macrofungal coculture systems will present broad applications.

## Author Contributions

GY and LT conceived and designed the review and wrote the main manuscript text, GY, YS, HH, XY, YW, XG, BQ, and LT searched the literatures and critically revised the manuscript. All authors contributed to the article and approved the submitted version.

## Conflict of Interest

The authors declare that the research was conducted in the absence of any commercial or financial relationships that could be construed as a potential conflict of interest.
